# Quality Characteristics and Volatile Profile of Macarons Modified with Walnut Oilcake By-Product

**DOI:** 10.3390/molecules25092214

**Published:** 2020-05-08

**Authors:** Anamaria Pop, Adriana Păucean, Sonia Ancuța Socaci, Ersilia Alexa, Simona Maria Man, Vlad Mureșan, Maria Simona Chiş, Liana Salanță, Iuliana Popescu, Adina Berbecea, Sevastiţa Muste

**Affiliations:** 1 Department of Food Engineering, Faculty of Food Science and Technology, University of Agricultural Sciences and Veterinary Medicine, 400372 Cluj-Napoca, Romania; anamaria.pop@usamvcluj.ro (A.P.); simona.man@usamvcluj.ro (S.M.M.); vlad.muresan@usamvcluj.ro (V.M.); simona.chis@usamvcluj.ro (M.S.C.); sevastita.muste@usamvcluj.ro (S.M.); 2 Department of Food Science, Faculty of Food Science and Technology, University of Agricultural Sciences and Veterinary Medicine, 400372 Cluj-Napoca, Romania; sonia.socaci@usamvcluj.ro (S.A.S.); liana.salanta@usamvcluj.ro (L.S.); 3 Department of Food Control, Faculty of Agro-Food Technologies, Banat University of Agricultural Sciences and Veterinary Medicine “King Michael I of Romania”, 300641 Timişoara, Romania; alexa.ersilia@yahoo.ro; 4 Department of Soil Sciences, Faculty of Agriculture, Banat University of Agricultural Sciences and Veterinary Medicine “King Michael I of Romania”, 300641 Timişoara, Romania; iuliapopescu2002@yahoo.com (I.P.); adinaberbecea@yahoo.com (A.B.)

**Keywords:** walnut oilcake, by-product utilization, macarons, quality characteristics, volatile profile, sensory evaluation

## Abstract

Walnut oilcake is a low-cost by-product of the edible oil industry but at the same time it is a valuable source of dietary fiber, natural antioxidants, and polyunsaturated fatty acids. In the context of health-friendly confectionary food products and to reduce the production cost, the aim of this study was to investigate the effect of walnut oilcake by-product on the quality characteristics and volatile profile of modified macarons. For this purpose, GC-MS and ITEX/GC-MS techniques were used to obtain the fatty acids methyl esters and the volatile profiles; physicochemical analyzes were performed to determine the nutritional characteristics and a nine-point hedonic scale test was performed for the sensory characteristics. The substitution of almond flour with 0%, 10%, 25% and 50% walnut oilcake powder increased the fiber, total phenolic content, and antioxidant capacity. Hedonic scores of the macaron samples made with different percentage of walnut oilcake decreased to additions of over 10%. Moreover, this result is emphasized by Pearson’s correlation parameters indicating as optimal addition for modified macarons, percentages up to 10% of walnut oilcake. This approach could reduce the costs related to the acquisition of the ingredients due to the oilcake price which is 3% of the almonds flour price.

## 1. Introduction

Macaron, a premium Western dessert, is small, circular shaped, crispy and moist textured cookie [1]. First produced in Italy, it is nowadays a luxury dessert loved all over the world through its association with French desserts [2,3] and it is also considered an expensive dessert [4]. The macaron has always been known as a cookie made only with sweet meringue and ground almond, characterized by a specific texture and taste, which increase the complexity of the sensorial perception. The addition of new ingredients can help to reformulate traditional recipes, providing countless creative possibilities [5].

Meringue is a basic baking product used to give light texture and bulk to desserts such as cakes and cookies. The role of meringues is very important for making macaroons as well [6]. When whipping the egg white quickly at a constant speed, the egg white protein forms bubbles. The sugar added to the whipped egg white absorbs moisture and forms a dense and shiny meringue [7].

Nowadays, food manufacturers (e.g., cookie factories and confectioneries) prefer ready-to-use liquid egg products to whole eggs. These products are more favorable because the user does not have to deal with breakage or storage of egg shells potentially infected with feces [8,9].

Food innovation can be considered as a process of combining ingredients using new techniques and/or novel raw materials. Innovations can also be a result of a combination of ingredients from existing recipes [10]. The process of innovation should account for the mastery of complexity where ingredients, texture, and taste merge to create this complexity with the purpose to bring significant and obvious benefit for consumers: it increases the variety of options available with a variation in combinations of ingredients, texture, taste, and product shapes and sizes [5].

Walnut oilcake flour is produced from walnut press-cake, a by-product from the cold pressing of walnut kernels and is considered a new product with potential use in the baking industry as a supplement or even as a replacement for wheat flour in the manufacture of bread, biscuits and cakes [11]. Its other use is to provide a source of natural favoring in these foods [12]. Other aspects to be considered in walnut oilcake valorization are recent changes in peoples’ dietary habits and consumers’ preference for food products without artificial additives (i.e., preservatives, artificial sweeteners, etc.) [9] The biggest challenge of the scientific world is to provide viable alternative models that combine food production with an efficient valorization strategy of waste and by-product, minimization of energy consumption, and environmental protection [13]. Oilcake from walnuts kernel is a rich source of protein and carbohydrates. Based on its particular technological properties, it is recommended to be used in the manufacture of vegetables, cereal products, sugary and flour-based confection foods [14]. Also, walnut press-cake is a rich source of bioactive compounds. Most of the phenolics in walnut are present in the defatted walnut matter and these antioxidant compounds remain in the walnut press-cake after cold-pressing. In addition, walnut press-cake has a high amount of tocopherol which has an important role in human health [11]. Walnuts are rich in both, linoleic acid and α-linolenic acid (ALA), the plant n-3 fatty acids. These compounds are important contributors to the human health due to their beneficial effects such as: protecting against the development of coronary heart disease and sudden cardiac death, lowering blood cholesterol, preservation or enhancement of low density lipoprotein (LDL) resistance to oxidation and improvement of endothelial function [15]. Walnuts are known to be a good source of essential linoleic acid, and therefore of polyunsaturated fatty acids (PUFA), which decreases LDL-cholesterol and increases HDL-cholesterol, moreover, they also contain phytosterols, dietary fiber, phenolics and tocopherols that may be beneficial for health [16,17,18,19]. The purpose of this study was to evaluate the influence of walnut oilcake powder (WP), on quality, textural profile, volatile profile and sensory characteristics of modified macarons in order to obtain pastry goods with-out food coloring and with improved quality characteristics. Walnut oilcake is used in most countries as fodder for animals and while the price varies from year to year due to the weather conditions, it remains cheaper than the almond flour, being the walnut oilcake price around 3% of the almond flour price. Through valorization of the walnut oilcake by-product, the cost with ingredients for produce modified macarons will be significantly reduced. Adding walnut oilcake by-product powder to the macarons will lead to improved quality of modified macarons, while simultaneously reducing costs production.

## 2. Results

### 2.1. The Chemical Composition and Mineral Profile of the Walnut Oilcake Powder

The approximate composition of the walnut oilcake powder (WP) is summarized in Table 1. The results are similar to those reported previously [12,14,19,20,21]. The residual oil content from WP is higher than reported values [14,19,20] which ranged between 6% and 18.2% and lower than 36.80% [11]. Similar values (20.3%) were also found [12]. Protein content in WP is lower than reported by several studies [11,12,14,19,20,21]. WP has high C18:2 and C18:3 contents and low saturated fatty acid/unsaturated fatty acid (SFAs/UFAs) value (Table 2). 

These results are aligned with reported data [22]. Walnut oilcake has high TPC and antioxidant capacity (Table 1), most of the phenolics in walnut are present in the defatted walnut matter and these antioxidant compounds remain in the oilcake after cold pressing [11]. The total phenolic content (TPC) was found in WP at the value of 136.33 mgGAE/100 g d.w. and radical scavenging activity value DPPH (53% inhibition). Similar results for by-products reused in the food industry have been obtained [23]; authors have reported that TPC values ranged from 133.93 mgGAE/100 g d.w. to 335.88 mgGAE/100 g d.w., and from 32.74% to 57.87% for radical scavenging activity, respectively. Regarding the nutritional value, results have shown that potassium, phosphorus, magnesium, and calcium had the highest levels of concentration for walnuts oilcakes, 506.88 mg/100 g, 267.37 mg/100 g and 128.24 mg/100 g, respectively (Table 1). Generally, the mineral content, according to their amount/100 g of product, was as follows: K > P > Mg > Ca > Fe > Zn > Mn > Cu. Our results were similar compared to the mineral values reported for Moldavian walnut oilcake [24], using the same method of analysis The sequence of mineral contents in Moldavian walnut oilcake products was K > Mg > Ca > Fe > Zn > Cu > Na, except the P, Mn that were not detected, compared to Romanian walnut oilcake. These differences of mineral content may be due to growth conditions, varieties, genetic factors, harvesting time, soil properties, geographical variations and analytical procedures [25,26].

### 2.2. Fatty Acids Methyl Esters Content and Volatile Compounds of Walnut Oilcake Powder

By means of the ITEX/GC-MS technique, a total of 19 volatile compounds was separated from the walnut oil cake powder on a ZB-5MS capillary column, of which 18, comprising two alcohols, two aldehydes, one ketone, five terpenes, three acids, two esters and other compounds like toluene were identified based on their mass spectra (Table 2).

Among the ketone class in WP, 1-phenylethanone is characterized by a sweet, floral and almond-like aroma [27,28]. 1-Phenylethanone was identified in all samples and non-significant differences between the macaron samples were recorded (*p* > 0.05, Table 2). Another compound with perceived almond and burnt sugar flavor was benzaldehyde. This would contribute to maintaining an aroma similar to that achieved by classic macarons. Most of the isolated and identified volatile compounds have pleasant aromas (sweet, fruity, herbal) and therefore the addition of WP may not negatively contribute to the final aroma of macarons.

### 2.3. The Effect of Walnut Oilcake Addition on Physicochemical Composition and Nutritional Quality of Modified Macarons

#### 2.3.1. Chemical Composition for Modified Macarons with Walnut Oilcake and Their Textural Profile Analyses

The proximate composition of the macarons with walnut oilcake (M0, M10, M25, M50) is summarized in Table 3. Incorporation of the WP in macarons has a significant influence on the major components (*p* < 0.05), except for protein and oil content.

The increase in moisture content from 6.34% for the control sample to 10.63% for 50% WP sample can be explained by the increasing of the fiber content which leads to higher water absorption during meringue preparation. Contrariwise, total carbohydrates amount decreased (from 72.88% to 63.35%) as the content of WP in the samples increased.

The protein, ash content, and oil content recorded non-significant differences for all the macaron samples (*p* > 0.05), as showed in Table 3. A cheaper raw material source like walnut oilcake by-product does not significantly change the values of these nutritional parameters.

Taking into account that in recent years cellulose has been considered to be one of the major ingredients to develop products of functional purpose, oilcakes with increased content of cellulose could be introduced as a new ingredient rich in insoluble dietary fibers [14].

The energy content of food is essential in dealing with problems associated with normal nutrition, under nutrition and obesity [29]. The energy value of the macaron samples decreased significantly (*p* < 0.05) from 350.44 to 322.73 kcal, directly proportional to the addition of walnut oilcake. Sample W0 contained the highest value while sample W50 had the least. Consumption of low energy density foods is fundamental to many weight loss plans [29,30].

Regarding the total phenolic content (TPC) in samples with different substitution level of WP, ranged from 20.33 mg GAE/100 g. d.w. (M0) to 62.66 mg GAE/100 g. d.w. (M50) and the antioxidant capacity (DPPH) ranged from 31.04% (M0) to 49.97% (M50). This trend of increased TPC and antioxidant capacity values for the pastry products with addition of walnut oilcake have been recorded in other similar studies [14,31]. The TPC and antioxidant capacity of the macaron samples were increased significantly (*p* < 0.05) by substituting the almond powder with walnut oilcake. A strong positive linear correlation was found between walnut oilcake percentage and TPC (r = 0.984), walnut oilcake percentage and inhibition of DPPH radical (r = 0.988). The improved antioxidant properties of the macarons with walnut oilcake might be due to the phenolic content of walnut oilcake. The total phenolic content is greatly influenced by factors, such as temperature, precipitation, natural causes (infection, damage, and pests), fruit maturity stage, and storage [32].

The macaron’s texture is an important attribute for consumers. The textural parameters of the macaron are given in Table 3. The control sample recorded the highest value of hardness (276 ± 55 g), followed by M10 (261 ± 15 g), M25 (248 ± 38 g), and M50 (56 ± 12 g). As the amount of the walnut oilcake powder increased in the macaron composition, the hardness decreased significantly (*p* < 0.05). The decrease in hardness, probably resulted from lower fat content of walnut oilcake compared to the fat content of the almond flour [11]. The drastic decline of the hardness at 50% WP addition lead to the conclusion that addition of walnut oilcake in macarons formulation must be under this value. The work necessary to overcome the internal strength of bonds within the macarons, together with the work performed by the sample against the compressing force as it is being removed, were measured as total work. The values recorded for total work [mJ], as shown in Table 3, followed the same trend of decreasing values (*p* < 0.05), indicating that the addition of WP influenced drastically the internal strength of the bonds within the macarons samples—decreasing from 4.57 mJ (control sample M0) to 1.6 mJ (sample M50-50% WP).

#### 2.3.2. Mineral Content of Macarons with Walnut Oilcake

The mineral composition of macaron samples (M0, M10, M25, M50) is shown in Table 4. Micronutrients as minerals are also important components when considering the nutritional characteristic of a potential food ingredient [33]. The mineral content of macarons is provided by the almond flour and WP added in different percentages in their composition. The scientific literature states that walnuts and almonds are very rich sources of minerals [34,35,36,37]. Almonds are one of the most nutrient-rich forms of food available [31] having a high content in minerals, as follows: calcium (264 mg/100 g), magnesium (268 mg/100 g), phosphorus (484 mg/100 g), potassium (705 mg/100 g), zinc (3.08 mg/100 g), copper (1.00 mg/100 g) and manganese (2.28 mg/100 g). The high mineral content of the walnut oilcake powder and almonds powder (K, P, Mg, Ca, Fe, Zn) is reflected, as we expected, in the mineral content of macaron samples. Significant differences (*p* < 0.05) of mineral content were recorded between macaron samples with different content of WP (Table 4). Potassium, phosphorus, magnesium, and calcium quantity decreased in the macaron samples, with the addition of WP in percentage of 10%, 25% and 50% WP compared to the control (0% WP).

Pearson minerals’ correlation for elements like Fe, Cu, Mn, and Zn (1.00, 1.00, 0.998 and 0.971, respectively) showed strong positive relationship between the percentage of walnut powder addition in the final baked products. On the other side, negative strong Pearsons’ correlations were found for elements like P, Mg, Ca, Cd and Cr (−0.990, −0.988, −1.00, −0.951 and 0.989, respectively), showing that walnut powder addition decreased the final baked content on these minerals. The quantity of essential elements as Fe, Mn, Zn and Cu, allows the walnuts oil cake, as well as the almond flour, to be considered as an excellent source of bio-elements [35,36,37,38,39].

### 2.4. The Effect of Walnut Oilcake Addition on Fatty Acids Methyl Esters Content of Modified Macarons

In the macaron samples, a total of *nine* fatty acid methyl esters (FAMEs) were identified, out of which six were classified as being saturated (SFAs), one as MUFA, and two as PUFAs. The main compounds from the saturated group in WP were palmitic acid, reaching around of 5.06% of total fatty acids, followed by lauric acid and caprylic acid. Walnut oilcake powder has high C18:2 and C18:3 contents and low ratio of saturated/unsaturated fatty acid (SFA/UFA). These results are aligned with the reported data [11,22].

Among PUFAs, linoleic acid reached the highest value; it could be noticed that linoleic acid content increased significantly with the WP addition in macarons. According to the study by [40], MUFA have been demonstrated to improve pancreatic beta-cell function and regulation of postprandial glycemia and insulin sensitivity. PUFA may act on the central nervous system protecting neuronal and cell-signaling function and maintenance, useful adjuvants to prevent, delay or ameliorate a number of chronic diseases in older people. As it can be seen in Table 5, the proportion of linoleic acid, linolenic acid, total unsaturated fatty acid (UFA) and total polyunsaturated fatty acids (PUFA) of the macarons samples increased with the increased percentage of walnut oilcake powder (WP), at the same time the proportion of the palmitic acid in fatty acid composition decreased. The increment in the proportion of oleic acid was significant (*p* < 0.05).

In the present study of modified macarons, a decrease of the omega-6/omega-3 ratio can be observed; the decrement is directly proportional to the percentage of WP added in macaron samples ranging from 10.8 (M0) to 8.04 (M25). This result is due to the significant increment (*p* < 0.05) of omega-3 acids content from 2.53 in control sample to 4.51 in M25. The result is important in the conclusion’s light of different studies which clearly show an increase in the risk of obesity and cardiovascular diseases as the amount of omega-6 fatty acids and the omega-6/omega-3 ratio increase [41].

Macarons with 25% walnut press-cake powder had the highest linoleic acid (36.29%), linolenic acid (4.51%), and total UFA (98.19%) and the lowest SFAs/UFAs value (0.10). The results are in agreement with those obtained previously [11].

### 2.5. The Effect of Walnut Oilcake Addition on Volatile Compounds Content of Modified Macarons

Using the ITEX/GC-MS technique, in the present study of modified macarons samples, a total number of 24 volatile compounds were separated on the capillary column, out of which 23 were identified by their mass spectra. Of the identified compounds three were alcohols, two aldehydes, two ketones, twelve terpenes, one acid, two esters, all listed in Table 6, along with their characteristic odors [23,28,42,43].

According to [44] hexanal was the largest compound in different varieties walnut kernels, followed by 1-pentanol, pentanal, 1-hexanol and then 1-penten-3-ol and are important contributors to walnut aroma. In the present study a high amount of hexanal was identify in the sample with the largest quantity of WP addition (M50), with a significant difference (*p* < 0.05) comparing to control, being characterized by having an aldehydic, grassy, leafy, fruity, sweety flavor. According to [19] the profile of the studied volatile walnut oils consists of aldehydes (31–37%), acids (20–38%) and alcohols (16–36%) with different proportions depending on the cultivar and hexanal—related to green descriptor—is the major contributor within the aldehydes family.

From the ketone group, 1-(4-propan-2-ylphenyl) ethenone was the main volatile compound identified in the control sample, while 1-phenylethanone identified in all samples, recorded non- significant value between the macaron samples (*p* > 0.05), being characterized by having an almond, floral flavor. The quantity of these compounds decreased with the addition of WP.

α and β-pinene are important compounds used in food preservation, due to their antimicrobial activity. More than that, α-pinene has been reported to have antitumor activity on a concentration of 8 mg/L [45]. α and β-pinene were found in all samples, in a similar level.

Limonene is one of the most common terpenes in nature, having a pleasant citrus-like flavor. The studies evaluating its safety showed that it has a low toxicity, being also biologically active. Limonene has been reported having chemo-preventive activity against different types of cancer such as breast or colorectal ones at a concentration between 0.5 to 12 g/m^2^/day [28,46]. The amount of limonene could be correlated with the quality of walnut. Limonene, a typical terpene, was identified as main compound in walnut oil sample from cold press, according to Bail at al. [47]. In our samples, limonene was identified in both control sample and modified macarons samples as being the major volatile compound. The concentration of limonene increased when WP was added to macarons.

### 2.6. Pearson’s Correlation

A Pearson’s correlation was run to study influence of the walnut oilcake percentage addition on the fatty acids and volatile compounds concentrations in macarons (Supplementary Materials).

Linolenic, caproic, ∑PUFAs, ∑UFAs and linoleic n-6 recorded positive Pearson’s values (r = 0.990, r = 0.989, r = 0.981, and r = 0.980, respectively), showing that the addition of walnut powder strongly influence the amount of these compounds in the final baked goods. On the other side, the ∑SFAs/∑UFAs recorded a strong negative Pearson correlation (−0.999), showing that the addition of walnut powder decreased the ∑SFAs/∑UFA’s value. In contrast, from the volatile compounds only 1-phenylethanone recorded a strong negative Pearson correlation (r = −0.974), indicating that the addition of WP does not significantly influence the aroma profile of macarons and therefore, from this point of view, it can also be used to replace almond flour. Regarding the effect of walnut powder addition on the sensory analysis of macarons, texture and overall acceptability were highly negative influenced, showing Pearson’s values such as r = −0.971 and r = −0.971, respectively.

### 2.7. The Effect of Walnut Oilcake Addition on Sensory Analysis of Modified Macarons

Table 7 shows the scores for the hedonic test of macarons made by addition of walnut oilcake in different percentages. Scores for appearance (color), flavor, taste, texture, and overall acceptance were highest for M10 sample, but not-significant differences compared to the control sample (*p* > 0.05) were found. The highest score for flavor was achieved by sample M25 (8.53) but the result was not significant (*p* < 0.05) compared to the control sample M0 and the sample M10.

This could be explained as a result of the presence of aroma volatile compounds such as α-pinene (perceived like fresh, sweet, green, woody and earthy flavor), β-pinene (perceived like pine and resin flavor), *p*-cymene (perceived like citrus, sweet, herbal and spicy flavor), limonene (perceived like citrus and mint flavor) and 1-methyl-4-(1-methylethenyl) benzene (perceived like spicy, balsamic and nutty, flavor). The perceived flavor may have a positive effect on flavor acceptability of macarons made with up to 25% addition of walnut oilcake powder. Meanwhile, macaron containing 50% WP, showed the lowest scores for flavor, taste, texture and overall acceptance (*p* < 0.05). This might be due to the excessive amounts of volatiles and phenolic compounds that can negatively alter the taste of the pastry food products. Similar results were reported previously on pastry products [11,48]. These results pointed out that a partial replacement of almond powder with up to 25% walnut oilcake in modified macaron increased the flavor of the product. These data suggest that addition of 10% WP is the optimal concentration for making modified macaron, for all sensory characteristics according to consumer preferences.

## 3. Materials and Methods

### 3.1. Supply of Raw Materials and Storage Conditions

Almond powder (50.6% fat, from which 3.9% was saturated fatty acids, 19.7% total carbohydrates from which 4.8% was sugars, 11.8% fiber, 21.3% protein), sugar powder, egg white, soybean protein isolate and granulated sugar were purchased from local specialized stores (DECO ITALIA SRL, Cluj-Napoca, România). According to the producers, all the ingredients meet the highest quality standards. The walnut residual cake, known under the brand name “LUNA SOLAI” was supplied by the TAF PRESOIL SRL company (Cluj, Romania), an important cold pressed oil processor from the Transylvania area [49]. Fresh walnuts, consisting of a range of different cultivars, were harvested from local orchards in July, 2017, and stored in their shells in open mesh bags at 15 °C until October 2017. The walnut oilcake by-product comes from the oil extraction processing of walnut kernels using a screw cold press (Komet Screw Oil, Expeller CA59G-of 6 mm of diameter and a screw speed of 30 rpm (IBG Monforts Oekotec GmbH & Co. KG, Mönchengladbach, Germany), and storage at 15 °C in sealing plastic containers for one week and then used in experiments. All reagents were of analytical grade. Analytical reagents and chemicals were purchased from Sigma Aldrich (St. Louis, MO, USA).

### 3.2. Walnut Oilcake Powder Preparation

The walnut oilcake by-product were passed through a mill feeder processed by grinding to fine flour (< 300 μm) on a Grindomix (Model GM200, Haan, Germany) laboratory mill at 10,000 rot/min for 50 s, passed through a 0.8 mm sieve and then homogenized by mixing. Walnut oilcake powder (WP) was used to substitute almond powder (AP) at different amounts (0–50%) as it could be seen in Table 8.

### 3.3. Modified Macarons Making Process

The formulation and the addition percentages of walnut oilcake powder were determined through preliminary industrial experiments and by using information from similar studies [3,50]. The modified macarons were prepared with the addition of oilcake powder at a weight percentage of 0% (M0), 10% (M10), 25% (M25) and 50% (M50) to the substitution of almond powder. The control sample (M0) was prepared with almond powder 100%. Before the macaron was prepared, the amount of Tant Pour Tant (TPT) was weighed, and the almond powder, walnut oilcake powder and sugar powder were dropped three times in a 30 mesh sieve, and the egg white-1 were mixed to make dough until the composition becomes the consistency of a paste. On the other hand, meringue was mixed with egg white-2, soybean protein isolate and citric acid, in a medium stage planetary mixer (Precise Heat Mixing Bowl, KitchenAid^®^, Greenville, OH, USA) for 2 min. The sugar and the water were boiled at a temperature of 117 °C and then the syrup was slowly poured into the mixed egg white to make a meringue by rotating quickly for 13 min, until the temperature drops to 40–42 °C. The finished dough is put in a pastry bag decorating tip couplers and squeezed in a dough tray with a constant weight and left at room temperature for 30 min to allow a crust to form. The macaroon batter was baked in an oven (Zanolli, Verona, Italy) preheated to 140 °C for 13 min with ventilation cooled in a refrigerator for 1 h and used in experiments. For the textural, moisture, volatile and sensorial profile, analyses were carried out on fresh macarons (on the day of baking) and for the rest of analyses the samples were kept in the freezer temperature.

### 3.4. Proximate Composition Analysis of the Walnut Oilcake Powder and Modified Macarons

The chemical analysis were carried out according to AACC Approved Methods [51]. Like moisture (44–15.02), lipids (30–25.01), ash (08–01.01), crude fibre (32–07.01) and protein were measured using the Kjeldahl method (46–11.02), nitrogen to protein conversion factor was 5.7. Total carbohydrate (%) content was calculated as the difference: 100 − (moisture + ash + proteins + lipids + crude fibres) equation reported in a previous work [21] and also reported by [28]. Energy value was estimated (kcal/100 g) by multiplying the percentage crude protein, crude lipid and carbohydrate by the recommended factor (2.44, 8.37 and 3.57 respectively) as described by Makinde and Adeyemi [29].

#### 3.4.1. Analysis of Macro and Microelements by Atomic Absorption Spectrophotometry

The analysis of macro and microelements was carried out according to SR EN 14082: 2003. Briefly, 3 g sample of mixed macarons were burned for 10 h at 650 °C in furnace (Nabertherm B150, Lilienthal, Germany). The ash was dissolved in HCl 20% and was transferred by a final volume of 20 mL in a volumetric flask. The macroelements (K, Ca, Mg) and microelements (Fe, Cu, Zn and Mn) were determined by atomic absorption spectroscopy (AAS; 220 FAA equipment, Varian, Mulgrave, Victoria, Australia). Mix standard solutions (ICP Multi Element Standard solution IV CertiPUR) were purchased from Merck, Bucharest, Romania All chemicals and solvents used in this study were of analytical grade. The detection limits (MDL) for analyzed elements were 0.02 ppm for Mg and K, 0.06 ppm for Fe, Cu, Zn, Mn, and Ca 0.03 ppm. The results were expressed as related to the macarons fresh weight.

#### 3.4.2. Total Phenolic Content (TPC) and Antioxidant Capacity

The methods described [11], Refs [52,53,54] were used with some modification, for total phenols and antioxidant capacity determination. Shortly, a 1-gram sample of mixed macarons was extracted three times with 100 mL acidified methanol (85:15 *v*/*v*, MeOH:HCl) by maceration under continuous stirring (Velp magnetic stirrer, Usmate, Italy) for 24 h. The filtrates were combined in a total extract, which was dried by using a vacuum rotary evaporator (KG—Laborota 4010 digital rotary evaporator, Heidolph Instruments GmbH & Co., Schwabach, Germany) at 40 °C. The dry residues were redissolved in 10 mL methanol (99.9% purity) and filtered through a 0.45 μm nylon filter (Millipore by Merck).

Total phenolic content (TPC) in phenolic extracts was determined by the Folin–Ciocalteu method. 100 μL of each extract was shaken for 1 min with 500 μL of Folin–Ciocalteu reagent and 6 mL of distilled water. After the mixture was shaken, 2 mL of 15% Na_2_CO_3_ was added and the mixture was shaken once again for 0.5 min. and water was added to reach a final volume of 10 mL. Samples were kept in the dark for 2 h, and then, absorbance was read at 720 nm on a Shimadzu 1700 UV/visible spectrophotometer (Shimadzu Scientific Instruments, Kyoto, Japan). The total phenol content was read by plotting the gallic acid calibration curve (from 1 to 1500 μg/mL) and expressed as milligrams of gallic acid equivalents (GAE) per gram of dry weight. The equation or the gallic acid calibration curve was y = 1.02295x + 0.08740; R^2^ = 0.99614.

Free radical-scavenging effects of phenolic extracts were measured according to [54,55]. Phenolic extracts of macaron samples were diluted with methanol at 0.5:99.5 ratio. Diluted phenolic extract (0.1 mL) was added to 3.9 mL of DPPH solution (0.025 g/L in methanol). The mixture was left for 30 min in the dark at room temperature. The absorbances of the sample at 515 nm were measured by a Shimadzu 1700 spectrophotometer against methanol as blank. Negative control was prepared using 0.1 mL methanol and 3.90 mL of DPPH. The inhibitory percentage of DPPH (2,2-diphenyl-1- picrylhydrazyl) was calculated according to the following equation:

% Inhibition = [(Ac − As)/Ac] × 100(1)

where Ac is the absorbance of the control (absorbance of DPPH solution), and As is the absorbance of the sample.

### 3.5. Determination of Fatty Acid Composition

#### 3.5.1. Total Lipid Determination

Lipids were extracted in petroleum ether using a Soxtest Raypa SX-6 MP (Barcelona, Spain) apparatus. Three g of crushed macaroons samples were introduced in cartridges and 50 mL petroleum ether was used for each sample extraction. Parameters were set as follows: temperature: 75 °C, time of extraction: 50 min. Samples were dried to constant weight. Total fat was expressed as percentage from the sample (% *w/w*).

#### 3.5.2. Fatty Acids Profile by GC-MS Analysis

Fatty acid methyl esters (FAMEs) from total lipids were analyzed using a Shimadzu GCMS-QP2010 PLUS apparatus and an AT-WAX column (30 m, 0.32 mm i.d., 1 µm thickness), (Shimadzu) belonging to the Interdisciplinary Research Platform of Banat University of Agricultural Sciences and Veterinary Medicine King Michael I of Romania. The solvent used for FAMEs was hexane, while the carrier gas (helium) was used at a flow rate of 1.00 mL/min and a linear velocity of 37.8 cm/s. Derivatization was performed for 1 h at 80 °C in an ultrasonic bath. An aliquot of 0.1 g sample was treated with 3 mL boron trifluoride methanol solution 20% (*w/v*). After cooling, 2.5 mL NaCl solution 10% (*w/v*) was added and methyl esters were extracted in 2 mL hexane, the organic layer being separated by centrifugation at 1006 G for 15 min using centrifuge Z36 HK (Hermle Labortechnik GmbH, Wehinge, Germany) The hexane solution (1 µL) was injected in apparatus. Samples were analyzed keeping the column initially at 140 °C for 10 min and then increasing the temperature with 7 °C /min up to 250 °C and maintaining at this temperature for 10 min (total run: 35.71 min). Split ratio was 1:10 and injection port temperature was set at 250 °C. The ion source and interface temperatures were 210 °C and 255 °C, respectively. FAME peaks were identified using NIST05 library and quantified by area normalization method. Some compounds were unidentifiable due to the lack of authentic samples and library spectra of the corresponding molecules. The percentage of various fatty acids was determined by reporting the peak area corresponding to a specific compound to the total peak area (for all identified constituents) of chromatograms [56].

### 3.6. Extraction and Analysis of Volatile Compounds by ITEX/GS-MS

The extraction of volatile compounds was performed using in-tube extraction (ITEX) technique coupled with their separation and identification by gas-chromatography hyphenated with mass spectrometry (GC-MS) [42]. Briefly, the extraction of volatile compounds was carried out by incubating at 60 °C for 20 min and under continuous agitation, a headspace vial containing 1 g of sample. After incubation the needle of the headspace syringe was introduced into the headspace of the vial and using the syringe plunger, the volatile compounds were adsorbed repeatedly (30 strokes) into a porous polymer fiber microtrap (ITEX-2TRAPTXTA, Tenax TA 80/100 mesh, ea). All the above operations as well the thermal desorption of the volatiles into the GC-MS injector were perform automatically by means of a CombiPAL AOC-5000 autosampler (CTC Analytics, Zwingen, Switzerland). The separation and identification of volatile compounds was carried out on a GC-MS QP-2010 (Shimadzu) model gas-chromatograph—mass spectrometer, using the method described by [47] with some modifications. The separation of volatile compounds was performed on a Zebron ZB-5 ms capillary column of 30 m × 0.25 mm i.d and 0.25 mm film thickness. The carrier gas was helium, 1 mL/min and the split ratio 1:20. The injector temperature, as well as those of ion source and interface were set at 250 °C. The temperature program of the column oven was: 38 °C (held for 5 min) increased to 110 °C at 4 °C/min and then to 250 °C at 20 °C/min (held for 5 min). The MS detection was performed on a quadrupole mass spectrometer operating in full scan (40–400 *m*/*z*) electron impact (EI) at ionization energy of 70 eV. The volatile constituents of the samples were identified based on their mass spectra, by comparison with those of reference compounds from NIST27 and NIST147 mass spectra libraries and verified with retention indices drawn from [57] or [58]. All peaks found in at least two of the three total ion chromatograms (TIC) were taken into account when calculating the total area of peaks (100%) and the relative areas of the volatile compounds [28,59].

### 3.7. Texture Profile Analysis for Macaron Samples

The texture of modified macarons with walnut oilcake powder (WP) was tested using a texture analyzer (CT 3 Texture Analyzer (Brookfield Engineering Labs, Middleboro, MA, USA) equipped with 10 kg load cell. Half macaron (crown or foot of the product) samples (cylindrical shape, 45 mm diameter and 12 mm height) were analyzed at room temperature. Texture profile analysis (TPA) was performed for each sample while using the TA41 cylindrical probe, 40% target deformation, 1 mm s^−1^ test and post-test speed, 5 g trigger load, and 5 s recovery time. However, due to the characteristic texture, none of the samples showed structure in the second cycle. Hardness and total work were computed by Texture Pro CT V1.6 software (Brookfield Engineering Labs).

### 3.8. Sensory Evaluation

The sensory analysis of the modified macaron sample was carried out by 50 semi-trained panelists (students and staff of Faculty of Food Science and Technology from Cluj-Napoca), 30% male and 70% female, age range: 20–55 years. Panelists were trained prior to evaluation to be familiar with quality attributes of macaron samples. Ethical approval was obtained from the scientific department before the study was conducted. The macarons were served one by one in a disposable plastic container (10 cm in diameter), marked with random three-digit number on the containing the sample so that there was no bias against the sample. The sensory test panel evaluated one sample so that the characteristic of each sample did not affect each other, and after rinsing the mouth with water, the next sample was evaluated, according to the method described by [3] with slight modifications, according to [28]. Each panelist analyzed three macaroons from each sample, taking into account the appearance, flavor, taste, texture and overall quality on a nine-point Hedonic scale, in the following sequence: 1–4 represent negative sensations, 5 was neither like nor dislike, and 6–9, which represent positive sensations, 9 meaning extremely like. The consumer preference is directly proportional to the score obtained (in the preference evaluation, the higher the preference, the higher the score).

### 3.9. Statistical Analysis

All experiments are performed in triplicate (n = 3). As indicated in the macaron making section, the effect of four percentage of walnut oilcake (0, 10, 25, and 50%) were analyzed. The obtained data were expressed as means ± standard deviations. Data were compared using Duncan multiple comparison test by using SPSS version 19 software (*p* ≤ 0.005) (IBM Corp., Armonk, NY, USA).

Pearson correlation was computed on Minitab 19.1 (Minitab Inc., State College, PA, USA) at 95 confidence level, in order to better analyze the influence of WP percentage addition on the macarons’ quality characteristics.

## 4. Conclusions

To the best of our knowledge, there are very few works reporting on the relationship between the walnut oilcake addition in baked goods and their fatty acids, volatile and sensory profiles. Walnut oilcake is a low-cost by-product of the oil industry and is a good source of valuable nutrients. The results of this study reveal the effect of walnut oilcake addition on physico-chemical parameters, fatty acids, and volatile profile of macarons with walnut oilcake. Pearson minerals’ correlation for Fe, Cu, Mn, and Zn showed strong positive relationship between the percentages of walnut oilcake added in the macarons. Also, linolenic, caproic, ∑PUFAs, ∑UFAs and linoleic n-6 recorded positive Pearson’s values, showing that the addition of walnut oilcake strongly influences the amount of these compounds in macarons. From the volatile compounds only 1-phenylethanone recorded a strong negative Pearson correlation, indicating that the addition of walnut oilcake does not significantly influence the aroma profile of macarons. Hedonic scores of the macaron samples made with different percentage of walnut oilcake decreased to additions of over 10%. Moreover, this result is emphasized by Pearson’s correlation parameters indicating as optimal addition for modified macarons, percentages up to 10% of walnut oilcake.

## Figures and Tables

**Table 1:** Chemical composition and mineral content of walnut oilcake powder.

Parameters	(WP)
**Proximate composition, % f.w.**	
Moisture content,	5.95 ± 0.57
Protein,	10.30 ± 0.28
Oil,	21.93 ± 0.19
Crude fiber,	6.79 ± 0.28
Ash,	5.28 ± 0.13
Carbohydrates,	49.75 ± 0.74
TPC, mgGAE/100 g dw.	136.33 ± 4.04
DPPH, %	53.00 ± 2.65
Minerals, mg/100 g f.w.	
K	506.88 ± 0.20
P	267.37 ± 0.47
Mg	128.24 ± 0.85
Ca	105.30 ± 1.11
Fe	2.98 ± 0.18
Zn	2.29 ± 0.14
Mn	1.77 ± 0.25
Cu	1.70 ± 0.21
Cd	0.070 ± 0.01
Cr	0.034 ± 0.01
Ni	0.18 ± 0.04

All determinations were performed in triplicate; f.w.—fresh weight; d.w.—dry weight.

**Table 2:** Fatty acids and volatile profile composition of walnut oilcake powder (WP).

**Fatty Acids**			**(WP)**
**Shorthand Nomenclature**	**Fatty Acid Name**	**Type**	**(%)**
C 6:0	Caproic	SFA	0.29 ± 0.04
C 8:0	Caprylic	SFA	0.60 ± 0.03
10:0	Capric	SFA	0.11 ± 0.06
12:0	Lauric	SFA	1.02 ± 0.12
14:0	Myristic	SFA	0.22 ± 0.06
16:0	Palmitic	SFA	5.06 ± 0.32
18:1(n-9)	Oleic	MUFA, ω-9	30.20 ± 0.98
18:3(n-3)	Linolenic	PUFA ω-3	10.64 ± 0.53
18:2(n-6)	Linoleic	PUFA ω-3	54.43 ± 0.49
∑SFAs	-	-	7.30 ± 0.17
∑PUFAs	-	-	65.07 ± 0.13
∑UFAs	-	-	95.27 ± 0.54
∑SFAs/∑ UFAs n-6/n-3	- -	- -	0.07 ± 0.04 5.11 ± 0.42
**Volatile profile**			(WP)
**Volatile Compounds**	**RI (Retention Indices)**	Characteristic Odour *	**Conc. (% of Total Peak Area)**
**Alcohols**			
Pentan-1-ol	759	Malty, alcoholic whiskey	0.92 ± 0.04
Hexan-1-ol	851	Ethereal, oil, alcohol, green, fruity, sweet, woody, floral	0.23 ± 0.05
**Aldehyde**			
Hexanal	801	Fresh, green, fatty, aldehydic, grass, leafy, fruity, sweaty	9.69 ± 0.03
Benzaldehyde	958	Almond, burnt sugar	0.53 ± 0.12
**Ketone**			
1-Phenylethanone	1042	Almond, floral	1.61 ± 0.09
**Terpenes and Terpenoids**			
α-Pinene	939	Fresh, sweet, green, woody, earthy	0.42 ± 0.06
β-Pinene	982	Pine, resin, turpentine	9.20 ± 0.77
β-Myrcene	992	Tropical, fruity with mango shades, grassy	1.45 ± 0.07
*p*-Cymene	1028	Citrus, sweet, herbal, spicy	0.41 ± 0.05
d-Limonene	1031	Citrus, mint	11.02 ± 0.20
**Acids**			
Benzoic Acid	1277	Balsamic	0.94 ± 0.05
**Esters**			
2-Methylpropyl acetate	778	Fruity, flowery, banana, pear	19.93 ± 0.95
Pentyl acetate	916	Herbal	0.38 ± 0.06
Methyl hexanoate	1000	Fruity, fresh, sweet	0.28 ± 0.04
Ethyl hexanoate	1001	Apple, fruity	11.55 ± 0.08
Hexyl acetate	1012	Fruity, herbal	23.73 ± 0.07
Ethyl octanoate	1042	Orange	3.87 ± 0.07
**Others**			
Nonane	900	Alkane	3.64 ± 0.10
N.D.	-	-	0.18 ± 0.02

All determinations were performed in triplicate * drawn from data base mentioned in Section 3.6.

**Table 3:** Chemical composition for modified macarons with walnut oilcake and their textural profile analyses.

Parameters	M0 (Control)	M10	M25	M50
**Proximate composition, % f.w.**				
**Moisture content**	6.34 ± 0.50 ^a^	7.16 ± 0.31 ^b^	8.08 ± 0.23 ^c^	10.63 ± 0.42 ^d^
**Protein**	7.13 ± 0.27 ^a^	7.30 ± 0.20 ^a^	7.81 ± 0.26 ^a^	7.61 ± 0.14 ^a^
**Oil**	8.74 ± 0.40 ^a^	8.97 ± 0.13 ^a^	8.85 ± 0.45 ^a^	9.03 ± 0.10 ^a^
**Crude fiber**	4.38 ± 055 ^a^	4.86 ± 0.21 ^ab^	5.79 ± 0.32 ^c^	6.79 ± 0.40 ^d^
**Ash**	1.67 ± 0.25 ^a^	1.73 ± 0.33 ^a^	1. 88 ± 0.28 ^a^	1.91 ± 0.07 ^a^
**Carbohydrates**	72.88 ± 0.35 ^d^	69.98 ± 1.15 ^c^	68.06 ± 0.66 ^b^	64.03 ± 0.28 ^a^
**Energy, kcal/100 g**	350.44 ^d^	342.71 ^c^	336.10 ^b^	322.73 ^a^
**TPC, mgGAE/100 g d.w.**	20.33 ± 2.40 ^a^	35.66 ± 4.93 ^b^	42.33 ± 3.06 ^c^	62.66 ± 4.03 ^d^
**DPPH, %**	31.04 ± 2.62 ^a^	35.06 ± 3.52 ^b^	38.10 ± 1.51 ^c^	49.97 ± 2.53 ^d^
**Texture parameters**				
**Hardness, g**	276 ± 55 ^d^	261 ± 15 ^c^	248 ± 38 ^b^	56 ± 12 ^a^
**Total work, mJ**	4.57 ± 1 ^b^	4.5 ± 1.3 ^b^	4.5 ± 1.2 ^b^	1.6 ± 0.6 ^a^

Values expressed are means ± standard deviation; M0 = 0% walnut oilcake powder supplemented almond powder (control sample); M10 = 10% walnut oilcake powder supplemented almond powder; M25 = 25% walnut oilcake powder supplemented almond powder; M50 = 50% walnut oilcake powder supplemented almond powder; f.w.—fresh weight; d.w.—dry weight; a-d Mean values in the same column with different superscript letters differ significantly (*p* < 0.05).

**Table 4:** Mineral content of modified macaron with walnut oilcake.

Samples	K	P	Mg	Ca	Fe	Zn	Mn	Cu	Cd	Cr	Ni
mg/100 g f.w.											
**M0 (Control)**	577.49 ± 0.53 ^d^	363.19 ± 0.16 ^d^	206.24 ± 0.80 ^d^	201.74 ± 0.45 ^d^	2.79 ± 0.59 ^a^	2.07 ± 0.04 ^a^	1.71 ± 0.51 ^a^	0.73 ± 0.12 ^a^	0.083 ± 0.01 ^a^	0.039 ± 0.02 ^a^	0.19 ± 0.03 ^a^
**M10**	575.32 ± 0.11 ^c^	361.75 ± 0.45 ^c^	202.17 ± 0.26 ^c^	197.12 ± 0.23 ^c^	2.87 ± 0.35 ^b^	2.35 ± 0.38 ^b^	1.85 ± 0.31 ^b^	0.78 ± 0.21 ^a^	0.077 ± 0.03 ^a^	0.037 ± 0.03 ^a^	0.17 ± 0.04 ^a^
**M25**	573.26 ± 0.19 ^b^	359.12 ± 0.44 ^b^	197.03 ± 0.40 ^b^	190.08 ± 0.93 ^b^	3.01 ± 0.32 ^c^	2.78 ± 0.13 ^c^	1.99 ± 0.43 ^c^	0.85 ± 0.18 ^ab^	0.067 ± 0.03 ^a^	0.036 ± 0.02 ^a^	0.16 ± 0.04 ^a^
**M50**	540.67 ± 0.35 ^a^	356.73 ± 0.80 ^a^	191.82 ± 0.48 ^a^	179.07 ± 0.27 ^a^	3.25 ± 0.42 ^d^	3.05 ± 0.66 ^d^	2.27 ± 0.38 ^b^	0.96 ± 0.31 ^b^	0.063 ± 0.03 ^a^	0.033 ± 0.01 ^a^	0.15 ± 0.04 ^a^

Values expressed are means ± standard deviation; M0 = 0% walnut oilcake powder supplemented almond powder (control sample); M10 = 10% walnut oilcake powder supplemented almond powder; M25 = 25% walnut oilcake powder supplemented almond powder; M50 = 50% walnut oilcake powder supplemented almond powder; f.w.—fresh weight; a-d Mean values in the same column with different superscript letters differ significantly (*p* < 0.05).

**Table 5:** Fatty acids methyl esters content (% of total fatty acids methyl esters) of macaron samples.

Shorthand Nomenclature	Fatty Acid Name	Type	M0 (Control)	M10	M25	M50
6:0	Caproic	SFA	0.12 ± 0.04 ^a^	0.14 ± 0.04 ^a^	0.23 ± 0.05 ^ab^	0.31 ± 0.06 ^b^
8:0	Caprylic	SFA	0.03 ± 0.03 ^a^	0.18 ± 0.03 ^b^	0.21 ± 0.08 ^ab^	0.28 ± 0.08 ^b^
10:0	Capric	SFA	0.04 ± 0.03 ^a^	0.08 ± 0.05 ^a^	0.16 ± 0.06 ^a^	0.18 ± 0.05 ^a^
12:0	Lauric	SFA	0.12 ± 0.06 ^a^	0.62 ± 0.11 ^b^	0.14 ± 0.06 ^a^	0.04 ± 0.03 ^a^
14:0	Myristic	SFA	0.18 ± 0.05 ^a^	0.61 ± 0.15 ^d^	0.46 ± 0.07 ^c^	0.31 ± 0.06 ^b^
16:0	Palmitic	SFA	11.96 ± 0.65 ^c^	11.83 ± 0.20 ^c^	11.50 ± 0.50 ^b^	9.24 ± 0.21 ^a^
18:1(n-9)	Oleic	MUFA, ω-9	43.29 ± 0.73 ^a^	52.47 ± 0.50 ^b^	56.77 ± 0.35 ^c^	58.11 ± 0.65 ^d^
18:3(n-3)	Linolenic	PUFA ω-3	2.53 ± 0.17 ^a^	3.06 ± 0.11 ^b^	3.35 ± 0.38 ^b^	4.51 ± 0.18 ^c^
18:2(n-6)	Linoleic	PUFA, ω−6	25.35 ± 0.44 ^a^	25.75 ± 0.59 ^a^	28.46 ± 0.49 ^b^	36.29 ± 0.44 ^c^
∑SFAs	-	-	12.45 ± 0.49 ^b^	13.46 ± 0.37 ^c^	12.70 ± 0.55 ^b^	10.36 ± 0.30 ^a^
∑PUFAs	-	-	27.88 ± 0.62 ^a^	28.81 ± 0.23 ^b^	31.81 ± 0.21 ^c^	40.08 ± 0.38 ^d^
∑UFAs	-	-	71.17 ± 0.16 ^a^	81.28 ± 0.73 ^b^	87.58 ± 0.26 ^c^	98.19 ± 0.62 ^d^
∑SFAs/∑UFAs	-	-	0.17 ± 0.04 ^a^	0.16 ± 0.04 ^a^	0.14 ± 0.05 ^a^	0.10 ± 0.03 ^a^
n-6/n-3	-	-	10.80 ± 0.28 ^c^	8.41 ± 0.37 ^b^	8.49 ± 0.15 ^b^	8.04 ± 0.61 ^a^

Note: Values expressed are means ± standard deviation of three replicates; WP = Walnut oilcake powder; M0 = 0% walnut oilcake powder supplemented almond powder (control); M10 = 10% walnut oilcake powder supplemented almond powder; M25 = 25% walnut oilcake powder supplemented almond powder; M50 = 50% walnut oilcake powder supplemented almond powder; FA—fatty acid; SFA—saturated fatty acids; PUFA—polyunsaturated fatty acids; MUFA—monounsaturated fatty acids; SFA/UFA—saturated/unsaturated fatty acid; UFA—unsaturated fatty acid; ∑UFA = MUFA + ∑PUFA. a-d Mean values in the same column with different superscript letters differ significantly (*p* < 0.05), according to Duncan’s multiple range test.

**Table 6:** Volatile compounds content of the modified macarons with walnut oilcake.

Volatile Compounds	RI (Retention Indices)	Characteristic Odor *	M0 (Control)	M10	M25	M50
**Alcohols**						
3-Methylbutan-1-ol	736	Malty, alcoholic whiskey,	5.33 ± 0.30 ^c^	0.59 ± 0.04 ^a^	2.53 ± 0.16 ^b^	2.51 ± 0.17 ^b^
Pentan-1-ol	759	Fruity, sweet,	-	-	-	0.35 ± 0.14
Hexan-1-ol	851	Ethereal, oil, alcohol, green, fruity, sweet, woody, floral	0.95 ± 0.08 ^a^	-	0.66 ± 0.05 ^a^	0.82 ± 0.11 ^a^
**Aldehyde**						
Hexanal	801	Fresh, green, fatty, aldehydic, grass, leafy, fruity, sweaty	2.43 ± 0.11 ^b^	1.84 ± 0.10 ^a^	2.45 ± 0.18 ^b^	4.09 ± 0.22 ^c^
Benzaldehyde	960	Almond, string, sharp, sweet, bitter, cherry	4.42 ± 0.15 ^bc^	4.11 ± 0.21 ^ab^	4.88 ± 0.13 ^c^	3.63 ± 0.35 ^a^
**Ketone**						
1-(4-Propan-2-yl- phenyl)ethanone	895	Fruity, spicy, sweet herbal, coconut woody	6.33 ± 0.16	-	-	-
1-Phenylethanone	1042	Almond, floral	0.27 ± 0.08 ^a^	0.25 ± 0.08 ^a^	0.24 ± 0.03 ^a^	0.17 ± 0.04 ^a^
**Terpenes and terpenoids**						
α-Pinene	939	Fresh, sweet, green, woody, earthy	1.64 ± 0.04 ^ab^	2.10 ± 0.24 ^b^	1.34 ± 0.35 ^a^	1.49 ± 0.15 ^a^
β-Pinene	982	Pine, resin, turpentine	7.01 ± 0.19 ^b^	9.90 ± 0.14 ^c^	5.77 ± 0.13 ^a^	6.19 ± 0.21 ^a^
β-Myrcene	992	Tropical, fruity with mango shades, grassy	7.98 ± 0.12 ^a^	8.52 ± 0.13 ^b^	8.45 ± 0.07 ^b^	8.82 ± 0.20 ^c^
α-Phellandrene	1006	Mint, spicy, citrus, fruity, herbal	1.56 ± 0.08 ^a^	1.83 ± 0.04 ^b^	1.95 ± 0.10 ^bc^	2.08 ± 0.15 ^c^
β-Phellandrene	1051	Mint, fruity, herbal, turpentine	14.37 ± 0.13 ^a^	18.35 ± 0.16 ^b^	19.08 ± 0.09 ^c^	19.29 ± 0.06 ^c^
*p*-Cymene	1028	Citrus, sweet, herbal, spicy	3.05 ± 0.10 ^a^	3.78 ± 0.04 ^b^	3.68 ± 0.11 ^b^	3.49 ± 0.06 ^ab^
*Trans*-β-Ocimene	1037	Sweet, herbal	1.02 ± 0.04 ^a^	1.41 ± 0.05 ^ab^	1.66 ± 0.06 ^b^	1.16 ± 0.09 ^ab^
d-Limonene	1031	Citrus, mint	36.24 ± 0.13 ^a^	39.94 ± 0.62 ^d^	37.43 ± 0.10 ^b^	38.37 ± 0.06 ^c^
β-Terpinene	1074	Citrus, tropical, fruity, oily, woody	1.63 ± 0.05 ^ab^	2.16 ± 0.08 ^b^	1.44 ± 0.08 ^a^	1.52 ± 0.10 ^a^
γ-Terpinene	1074	Citrus, tropical, fruity, oily, woody	1.53 ± 0.05 ^a^	1.73 ± 0.10 ^a^	3.02 ± 0.13 ^b^	1.63 ± 0.12 ^a^
1,3,8-*p*-Mentha- triene	1110	Woody, citrus, grassy	-	0.60 ± 0.08 ^a^	1.21 ± 0.08 ^b^	0.75 ± 0.11 ^ab^
1-Methyl-4-(1-methyl- ethenyl)benzene,	1284	Spicy, balsamic, nutty, terpenic	0.88 ± 0.04 ^ab^	0.43 ± 0.08 ^a^	1.28 ± 0.10 ^b^	0.79 ± 0.09 ^ab^
**Acids**						
Benzoic acid	1277	Balsamic	1.27 ± 0.09 ^a^	0.91 ± 0.07 ^a^	2.10 ± 0.10 ^b^	0.70 ± 0.03 ^a^
**Esters**						
Ethyl hexanoate	1001	Fruity, sweet, apple	0.53 ± 0.04	-	-	-
Hexyl acetate	1011	Fruity, spicy, herbal, citrus, green	1.14 ± 0.08 ^b^	0.54 ± 0.11 ^a^	0.73 ± 0.05 ^a^	1.15 ± 0.08 ^b^
**Others**						
Toluene	775	Solvent	0.42 ± 0.07 ^a^	0.33 ± 0.10 ^a^	0.43 ± 0.06 ^a^	0.30 ± 0.02 ^a^
N.D.	-	-	-	0.67 ± 0.05 ^a^	1.07 ± 0.08 ^b^	0.75 ± 0.09 ^a^

Note: ^a–f^ different superscripts in a row indicate significant difference within samples (*p* < 0.05). All determinations were performed in triplicate N.D.—not detected; * drawn from data base mentioned in Section 3.6.

**Table 7:** Sensory evaluation of modified macaron samples.

Macaron Samples	Appearance	Flavor	Taste	Texture	Overall Acceptability
**M0 (Control)**	8.40 ± 0.42 ^bc^	8.18 ± 0.20 ^b^	8.05 ± 0.35 ^c^	8,63 ± 0.39 ^c^	8.30 ± 0.57 ^c^
**M10**	8.58 ± 0.46 ^c^	8.40 ± 0.14 ^b^	8.20 ± 0.42 ^c^	8.78 ± 0.18 ^c^	8.40 ± 0.42 ^c^
**M25**	8.10 ± 0.71 ^b^	8.53 ± 0.39 ^b^	7.23 ± 0.11 ^b^	7.65 ± 0.21 ^b^	7.65 ± 0.64 ^b^
**M50**	7.20 ± 0.14 ^a^	6.38 ± 0.31 ^a^	6.90 ± 1.14 ^a^	6.03 ± 0.53 ^a^	6.70 ± 0.28 ^a^

Values expressed are means ± standard deviation; M0 = 0% walnut oilcake powder supplemented almond powder (Control); M10 = 10% walnut oilcake powder supplemented almond powder; M25 = 25% walnut oilcake powder supplemented almond powder; M50 = 50% walnut oilcake powder supplemented almond powder; a-d Mean values in the same column with different superscript letters differ significantly (*p* < 0.05).

**Table 8:** Formulation used for the preparation of modified macarons.

Mixing Ratio	Components (g)	M0 (Control)	M10	M25	M50
Tant pour tant (TPT)	Almond powder (AP)	300	270	225	150
	Walnut oilcake powder (WP)	-	30	75	150
	Sugar powder	300	300	300	300
	Egg white-1	110	110	110	110
Meringue	Sugar	300	300	300	300
	Water	75	75	75	75
	Egg white-2	115	115	115	115
	Soybean protein isolate	10.2	10.2	10.2	10.2
	Citric acid	3.4	3.4	3.4	3.4

## Supplementary Materials

Table SM: Pearson Correlations—the influence of walnut powder addition on the macarons’ quality characteristics.
