# Supplementary material for: Quality Characteristics and Volatile Profile of Macarons Modified with Walnut Oilcake By-Product
Source: Molecules. 2020 May 8;25(9):2214. doi: 10.3390/molecules25092214 (PMC7249020; doi:10.3390/molecules25092214)
Supplement: Supplementary file 1 [file molecules-25-02214-s001.pdf]

Table SM - Pearson Correlations - the influence of walnut powder addition on the macarons' quality characteristics*

**Parameters**	**Correlation**	**P-Value**
**Proximate composition for macarons with walnat oilcake and their textural profile analyses**		
Moisture content,%	0.994	0.006
Protein, %	0.725	0.275
Fat, %	0.733	0.267
Crude fiber, %	0.999	0.001
Ash (%)	0.861	0.139
Carbohidrate, %	-0.991	0.009
TPC (mgGAE/100g)	0.984	0.016
DPPH (%)	0.988	0.012
Energy	-0.997	0.003
Hardness	-0.928	0.072
Total work	-0.890	0.110
**Minerals content of macaroons with walnut oilcake**		
K	-0.923	0.077
P	-0.990	0.010
Mg	-0.988	0.012
Ca	-1.000	0.000
Fe	1.000	0.000
Zn	0.971	0.029
Mn	0.998	0.002
Cu	1.000	0.000
Cd	-0.951	0.049
Cr	-0.989	0.011
Ni	-0.931	0.069
**Fatty acids methyl esters content content of the macarons with walnut oilcake**		
Caproic	0.989	0.011
Caprylic	0.898	0.102
Capric	0.934	0.066
Lauric	-0.457	0.543
Myristic	-0.004	0.996
Palmitic	-0.941	0.059
Oleic	0.854	0.146
Linolenic n-3	0.990	0.010
Linoleic n-6	0.971	0.029
∑SFAs	-0.815	0.185
∑PUFAs	0.981	0.019
∑UFAs	0.980	0.020
n-6/n-3	-0.744	0.256
∑SFAs/∑UFAs	-0.999	0.001
**Volatile compounds**		
**Alcohols**		
3-Methylbutan-1-ol	-0.300	0.700
Pentan-1-ol	0.881	0.119
Hexan-1-ol	0.213	0.787
**Aldehyde**		
Hexanal	0.863	0.137
Benzaldehyde	-0.510	0.490
**Ketone**		
1-(4-propan-2-ylphenyl) ethanone	-0.651	0.349
1-Phenylethanone	-0.974	0.026
**Terpens and Terpenoids**		
α-Pinene	-0.495	0.505
β-Pinene	-0.500	0.500
β-Myrcene	0.875	0.125
α-Phellandrene	0.925	0.075

β- Phellandrene	0.761	0.239
p-Cymene	0.322	0.678
Trans-β - Ocimene	0.111	0.889
D-Limonene	0.236	0.764
β-Terpinene	-0.465	0.535
γ-Terpinene	0.136	0.864
1,3,8-p-Menthatriene	0.594	0.406
Benzene,1-methyl-4-(1-methylethyl)	0.175	0.825
**Acids**		
Benzoic acid	-0.229	0.771
**Esters**		
Ethyl hexanoate	-0.651	0.349
Hexyl acetate	0.277	0.723
**Others**		
Toluene	-0.568	0.432
**Sensory evaluation of macaron samples**		
Appearance	-0.942	0.058
Flavor	-0.807	0.193
Taste	-0.921	0.079
Texture	-0.971	0.029
Overall acceptability	-0.971	0.029

* Statistically significant when P-value < 0.05
